# Gastric emphysema and pneumatosis intestinalis in two common marmosets with duodenal dilation syndrome

**DOI:** 10.1186/s12917-024-04087-8

**Published:** 2024-05-24

**Authors:** Shinpei Kawarai, Yasuhiro Sakai, Atsushi Iriki, Yumiko Yamazaki

**Affiliations:** 1 Laboratory for Symbolic Cognitive Development, RIKEN Center for Biosystems Dynamics Research, MI R&D Center Building 3F, 6-7-3 Minatojima-Minamimachi, Chuo-Ku, Kobe, Hyogo 650-0047 Japan; 2 Department of Veterinary Nursing for Companion Animals, Chuo Animal General Professional Training College, 1-12-17 Tsuji, Shimizu-ku, Shizuoka, Shizuoka 424-0806 Japan; 3 Department of Tumor Pathology, Hamamatsu University School of Medicine, 1-20-1 Handayama, Chuo-Ku, Hamamatsu, Shizuoka 431-3192 Japan; 4 RIKEN Innovation Design Office, 2-1 Hirosawa, Wako, Saitama 351-0198 Japan; 5 Department of Psychological Sciences, Niigata University of Health and Welfare, 1398 Shimami-Cho, Kita-Ku, Niigata, Niigata 950-3198 Japan

**Keywords:** Cause of death, Chronic vomiting, Common marmoset, Duodenal dilation, Gas cysts, Gastric emphysema, Gastrointestinal disease, Hepatic portal venous gas, Pneumatosis intestinalis

## Abstract

**Background:**

Common marmosets (*Callithrix jacchus*) are widely used as primate experimental models in biomedical research. Duodenal dilation with chronic vomiting in captive common marmosets is a recently described life-threatening syndrome that is problematic for health control. However, the pathogenesis and cause of death are not fully understood.

**Case presentation:**

We report two novel necropsy cases in which captive common marmosets were histopathologically diagnosed with gastric emphysema (GE) and pneumatosis intestinalis (PI). Marmoset duodenal dilation syndrome was confirmed in each case by clinical observation of chronic vomiting and by gross necropsy findings showing a dilated, gas-filled and fluid-filled descending duodenum that adhered to the ascending colon. A diagnosis of GE and PI was made on the basis of the bubble-like morphology of the gastric and intestinal mucosa, with histological examination revealing numerous vacuoles diffused throughout the lamina propria mucosae and submucosa. Immunostaining for prospero homeobox 1 and CD31 distinguished gas cysts from blood and lymph vessels. The presence of hepatic portal venous gas in case 1 and possible secondary bacteremia-related septic shock in case 2 were suggested to be acute life-threatening abdominal processes resulting from gastric emphysema and pneumatosis intestinalis.

**Conclusions:**

In both cases, the gross and histopathological findings of gas cysts in the GI tract walls matched the features of human GE and PI. These findings contribute to clarifying the cause of death in captive marmosets that have died of gastrointestinal diseases.

## Background

Common marmosets (*Callithrix jacchus*) are widely used as primate experimental models in biomedical researches [11]. Chronic recurrent gastrointestinal (GI) diseases are common in laboratory marmoset colonies and are sometimes becoming a life-threatening problem in health control.

Histopathological findings on necropsy have shown inflammatory bowel disease in the forms as chronic lymphocytic enteritis (CLE) and colitis [3, 10]. Duodenal lesions, such as strictures near the major duodenal papilla [17] and proximal duodenal obstruction and dilation (“marmoset duodenal dilation syndrome (MDDS)” [12]), are also considered significant causes of morbidity and mortality in laboratory marmosets [3]. Although many autopsy cases have been reported, further observation and analysis are required to determine the etiology of this disease.

An MDDS is a diagnosis of captive marmosets exhibiting repetitive vomiting, chronic bloating, and exhaustion [12]. The gross morphological criterion of MDDS consists of a dilated duodenum that is filled with a mixture of gas and fluid and whose descending part has a maximum diameter > 12 mm. Along with severe duodenitis, duodenal ulceration and fibrosis, and peritonitis, MDDS can cause adhesions between the duodenum and large intestine. Histopathological examination revealed CLE, cholangitis, cholecystitis, and pancreatitis in the affected marmosets. Multiple factors are suspected, as the etiology of MDDSs involves bacterial infection, ulceration, and fibrosis leading to stricture, as well as dietary factors such as overeating or consuming highly fermentable food; however, the causes of death have not been fully determined [3, 12].

In humans, gastric emphysema (GE) and pneumatosis intestinalis (PI) occur in the GI tract wall through the accumulation of gas from outside the GI tract or the production of gas within the GI wall [1, 6, 20]. Gross findings of bubble-like structures and histopathological findings of gas cysts are typical features of GE and PI [2, 6, 20]. A common clinical sign of GE is frequent vomiting [1, 5, 8], while those of PI are diarrhea, bloody stools, abdominal pain, constipation, weight loss, and tenesmus [19]. In PI, mucosal disruption allows invasion of the flatus and translocation of bacteria into the lesion [6, 19, 20], followed by the formation of hepatic portal venous gas (HPVG) and secondary bacteremia, which are life-threatening, acute abdominal processes [13–15, 19].

Here, we report two cases in which captive marmosets developed sudden hypothermia and heavy vomiting with hematemesis after long-term chronic vomiting. Gross and histopathological examination confirmed the diagnosis of MDDS, with concurrent GE and PI. To our knowledge, this is the first report of simultaneous MDDS, GE, and PI in the common marmoset.

## Case presentation

Case 1, male, 9 years and 5 months old, 291 g body weight (BW), was euthanized because of sudden severe hypothermia (34.1 °C), seizures, and moderate vomiting (approximately 4–6 ml) with a small amount of blood. It had been treated for 5 years and exhibited frequent vomiting that fluctuated between mild (< 3 ml) and heavy (> 7 ml). Case 1 showed changes in BW with vomiting and tested negative for infectious pathogens (Table 1). Case 2, female, 6 years and 11 months old, 353 g BW, died the day after sudden severe hypothermia (34.7 °C) and heavy vomiting with hematemesis. This marmoset had been treated for frequent, fluctuating, mild to moderate vomiting without persistent diarrhea or weight loss for 2 years, and the frequency of heavy vomiting without hematemesis increased 1 month before its death. Both animals were treated with drugs as needed (Table 1).

**Table 1:** Drugs and tests employed in cases 1 and 2

	Category	Name	Company (place)	case 1	case 2	Note
Drugs	Antibiotics	Baytril	Bayer Pharma Japan (Tokyo, Japan)	●		0.06 ml/head, s.c.,SID, continuous 5–7 days
	Fluid replacement	Lactec	Otsuka Pharmaceutical Factory, Inc. (Tokushima, Japan)	●	●	3.0—6.0 ml, s.c., SID, when heavily vomitted
	Drugs for GI motility	Primperan Injection 10 mg	Astellas Pharma, Inc. (Tokyo, Japan)	●		0.04 ml/head, s.c., SID, when vomitted
	H2-blockers	Famotidine	Nichi-Iko (Toyama, Japan)	●		0.06 ml/head, s.c., SID, everyday
Tests	Blood cell counts	Microsemi LC-662	Horiba, Ltd. (Kyoto, Japan)	●	●	blood sample
	Blood chemistry	Vet-scan VS2	Zoetis Japan (Tokyo, Japan)	●	●	blood sample
	*Clostridioides difficile*	Techlab C Diff Quick Chek Complete	Alere (Chiba, Japan)	(-)		fecal sample
	Giardia	SNAP Giardia Test	IDEXX (Maine, USA)	(-)		fecal sample
	Helicobacter species	Testmate Rapid Pylori Antigen	BD (Tokyo, Japan)	(-)		fecal sample

●: drugs/tests employed

(-): tested and got negative results

Serum biochemical tests (Table 2) and complete blood counts (CBCs; Table 3) were performed for both animals. The last serum biochemistry panel before euthanasia suggested chronic maldigestion (hypoalbuminemia) and acute renal injury (azotemia, markedly elevated creatinine, hyperglycemia, hypercalcemia, hyperphosphatemia, and low Na/K ratio) in case 1 and mild maldigestion (slight hypoalbuminemia, reduced blood urea nitrogen, and creatinine) in case 2 (Table 2). We did not perform any antemortem imaging.

**Table 2:** Serum biochemical profiles in cases 1 and 2

Cases, Age (year, month)		Case 1, 7y7m	Case 1, 9y5m	Case 2, 5y7m	Case 2, 6y9m	Reference interval from our laboratory^a^	Reference interval from reference 8^b^
	unit						
TP	g/dL	6.7	7.6	6.3	5.3	6.1 – 6.5	5.2 – 6.4
ALB	g/dL	3.6	1.3	3.6	2.3	3.1 – 3.5	3.1 – 4.0
GLOB	g/dL	3.2	6.3	2.7	3.0	2.1 – 4.3	ND
A/G ratio		1.1	0.2	1.3	0.8	1.1 – 1.2	ND
ALP	µkat/L	2.1	2.5	1.6	0.5	0.4 – 2.0	0.7 – 1.9
ALT	µkat/L	0.1	0.3	0.3	0.1	0.1 – 1.9	0.1 – 0.2
AMY	µkat/L	1.4	1.8	1.3	1.1	1.1 – 1.2	2.9 – 4.8
TBIL	µmol/L	5.1	5.1	6.8	5.1	3.4 – 8.5	1.7 – 3.4
BUN	mmol/L	1.1	45.0	25.0	0.7	7.6 – 8.4	5.1 – 9.1
CRE	µmol/L	35.4	636.5	44.2	8.8	28.0 – 35.2	8.8 – 35.4
GLU	mmol/L	7.7	19.3	9.3	8.7	5.2 – 14.3	3.9 – 8.6
Ca, total	mmol/L	2.2	2.6	2.0	2.2	2.1 – 2.2	1.9 – 2.6
PHOS	mmol/L	1.3	7.8	1.6	1.2	0.5 – 1.7	0.9 – 1.5
Na +	mmol/L	156.0	132.0	142.0	156.0	152.1 – 154.3	147.4 – 152.5
K +	mmol/L	4.4	10.6	2.8	4.8	4.3 – 4.6	2.7 – 3.5
Na/K ratio		35.5	12.5	50.7	32.5	33.7 – 35.7	ND

*TP* total protein, *ALB* albumin, *GLOB* globrin, *A/G ratio* alubimin/globrin ratio, *ALP* alkaline phosphatase, *ALT* alanine aminotransferase, *AMY* amylase, *TBIL* total bilirubin, *BUN* blood urea nitrogen, *CRE* creatinine, *GLU* glucose, *Ca total* calcium, total, *PHOS* phosphorus, *Na* + sodium;

K + : potassium; Na/K ratio: sodium/potassium ratio; ND: not described

^a^Data from our marmoset colony (adults [older than 2y] housed indoors; *n* = 21)

^b^Data for adult (older than 2y) common marmosets (*n* = 41) were obtained from ref [7]

**Table 3:** Complete blood count results in cases 1 and 2

Cases, Age (year, month)	Case 1, 7y7m	Case 2, 5y7m	Case 2, 6y9m	Reference interval from our laboratory^a^
White blood cell (× 10^9^/L)	6.1	7.1	6.4	4.4 – 7.3
Red blood cell count (× 10^12^/L)	5.97	7.28	5.1	4.8 – 6.1
Hemoglobin (g/l)	129	158	111	104.8 – 130.9
Hematocrit (%)	42.2	49	36.1	32.5 – 41.3
Mean corpuscular volume (fL)	70.7	67.4	70.7	66.6 – 69.4
Mean corpuscular hemoglobin (pg/cell)	21.6	21.8	21.7	21.3 – 22.3
Mean corpuscular hemoglobinconcentration (g/L)	306	323	307	316.4 – 325.7
Platelet count (× 10^9^/L)	1029	583	669	531.2—647.8

^a^Data from our marmoset colony (adults [older than 2y] housed indoors; *n* = 14)

**Fig. 1:**
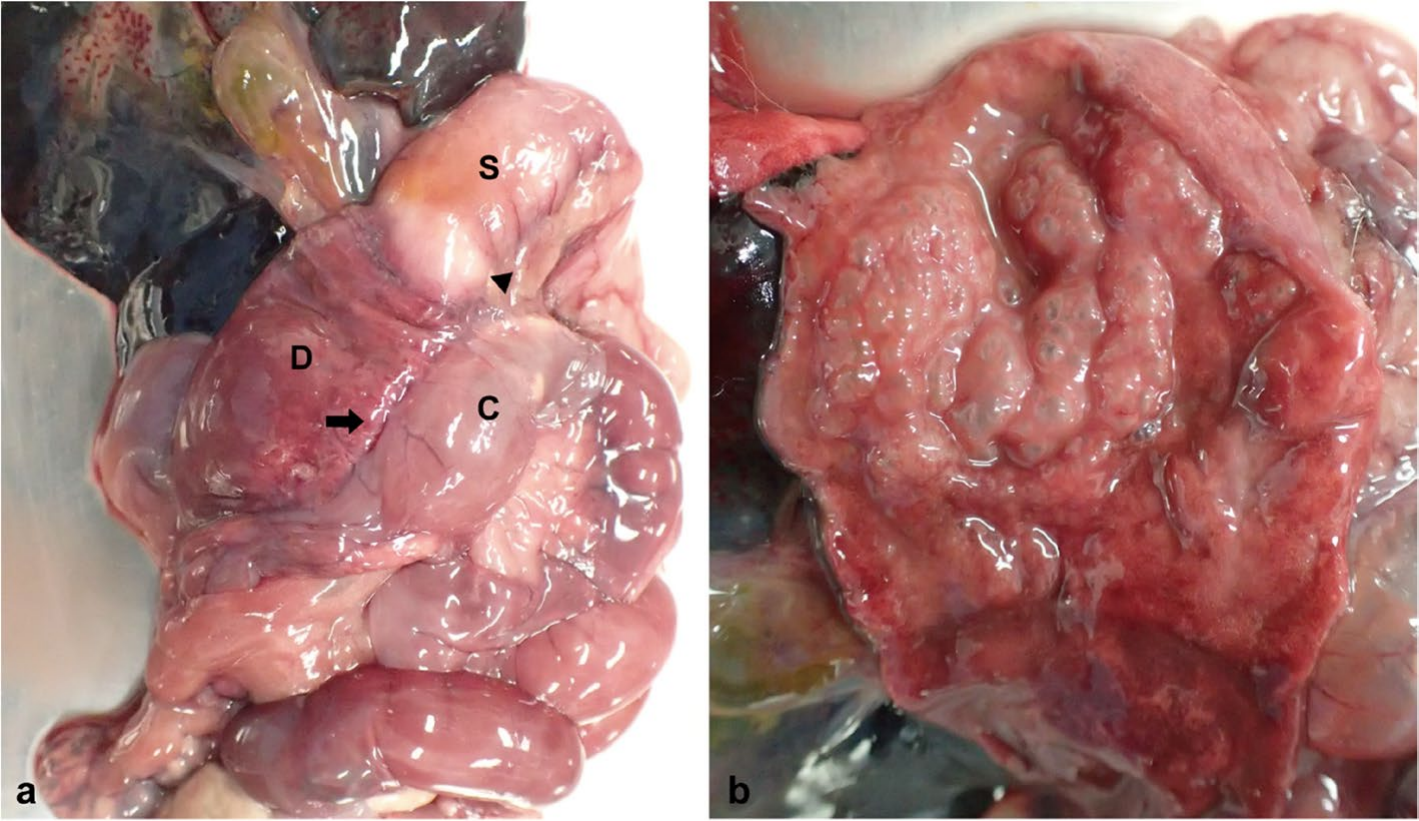
Marmoset duodenal dilation syndrome, case 2. **a** Marked dilation of the stomach (S) and duodenum (D). The ascending colon (C) tightly adheres to the stomach (arrowhead) and duodenum (arrow). The peritoneum between the colon and stomach is thickened by fibrosis. The abdominal organs are slightly pale reddish in color, indicating gastritis, and the GI tract is bloated. **b** Inside of the dilated stomach. A bloody exudate is retained. Odd-shaped, edematous gastric mucosa with diffuse numerous bubble-like structures (gas cysts characteristic of gastric emphysema)

The necropsy of case 1 was performed soon after euthanasia. The stomach and proximal to the descending parts of the duodenum were markedly dilated (18.3 mm in diameter at the proximal duodenum; reference interval of our laboratory (*n* = 7): 7.0 mm ± 1.6 mm), as measured before opening the lumen, accompanying intraabdominal fibrous adhesion among the ascending colon, stomach, and descending part of the duodenum. The necropsy of case 2 was performed the morning after she died. The diameter of the region proximal to the descending part of the duodenum was 21.0 mm, with fibrous adhesions formed among the stomach, duodenum, and colon (Fig. 1a, b). A sanguineous exudate was retained in the lumen of the dilated stomach and duodenum. The findings of odd-shaped, edematous gastric mucosa with diffuse numerous bubble-like structures (Fig. 1b) matched the typical features of human GE and PI [2, 6, 20]. Thus, in addition to the major symptoms of vomiting and hypoalbuminemia, the gross necropsy findings in both cases were compatible with the diagnostic criteria of MDDS [3, 12].

**Fig. 2:**
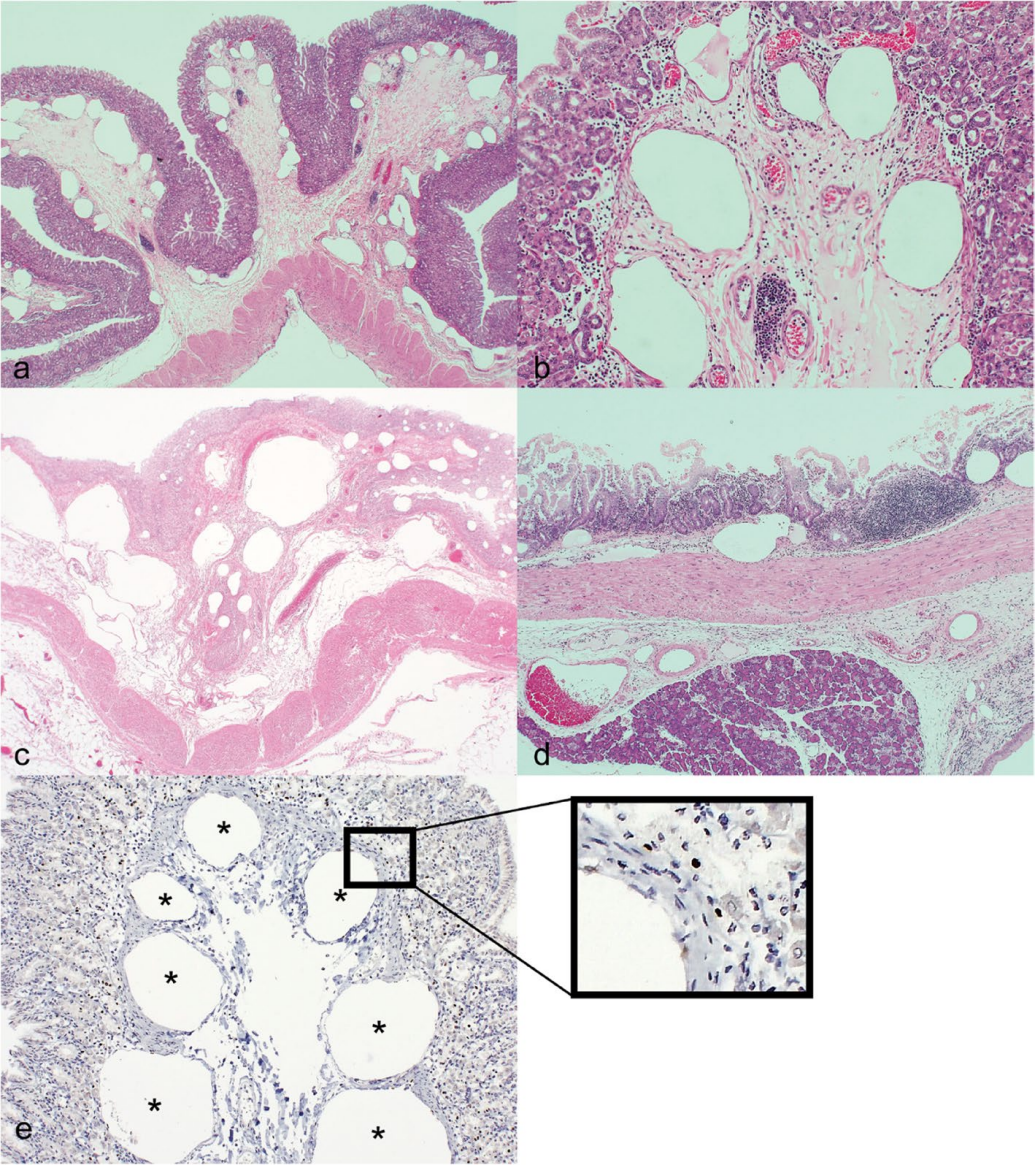
Gastric emphysema and pneumatosis intestinalis, marmoset, HE. **a**, **b** Stomach, case 1. **c** Stomach, case 2. **d** Duodenum, case 1. The gastric and duodenal mucosa contain numerous empty spaces, which are gas cysts. **e** Liver, case 1. Abnormal dilation of the portal vein suggests hepatic portal venous gas. **f** Liver, case 2. There are numerous rods, neutrophil infiltration, and liver cell necrosis around the main trunk of the portal vein

After gross examination, the tissues of both animals were immediately fixed in 10% neutral buffered formalin, and then paraffin-embedded 5-μm-thick staining sections were prepared at a commercial laboratory for histopathological examination. In case 1, numerous diffusely distributed vacuoles of various sizes were observed in the lamina progeria mucosae, submucosa, and subserosa of the stomach and duodenum (Fig. 2a,b, and d). Gastritis with ulcers reached the submucosa. Dilated and congested portal veins in Glisson’s capsules of the liver were also observed, which indicated HPVG (Fig. 2e).

**Fig. 3:**
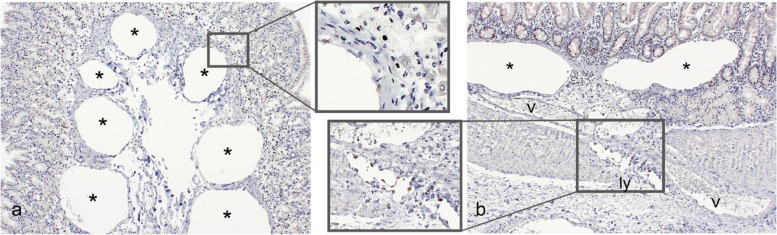
Gas cysts identified by immunohistochemistry, case 1. Immunohistochemistry was performed with a mouse anti-human CD31 monoclonal antibody (clone JC70A) and a mouse anti-human prospero homeobox 1 (Prox1) monoclonal antibody (clone 1D4B1). The amino acid sequences of human CD31 and Prox1 almost completely match those of *C. jacchus* CD31 (90%) and Prox1 (100%), as indicated by Standard Protein Blast (NIH, Bethesda, MD, USA). a) Stomach. b) Duodenum. Gas cysts (asterisk) are observed in the mucosa, independent of the blood and lymph vessels (v and ly, respectively). Note that the nuclei of the lymphatic endothelial cells as well as some of the enteric epithelial cells and pancreatic acinar cells are positive, but vascular endothelial cells and gas cyst walls are negative. The universal immunoenzyme polymerase method is visualized with DAB (Histofine; Nichirei, Tokyo, Japan)

In case 1, immunohistochemical staining for CD31 and prospero homeobox 1 (Prox1) was performed to distinguish the conditions from chronic lymphocytic enteritis, lymphangioma, and lymphangiectasis. The results showed that the walls of diffusely distributed vacuoles were mostly negative, and some vacuoles with positive endothelial cells were blood and lymph vessels (Fig. 3a, b). Thus, the vacuoles were gas cysts [6, 20] without an endothelium-lined cystic structure and were unlikely to have developed from lymph vessels.

Case 1 showed marked congestion of the liver (with centrilobular congestion and liver cell necrosis) and bilateral kidneys. Mild chronic hepatitis with slight fibrosis, hemosiderin deposition in the liver, and acute and chronic cholecystitis, which are common histopathological findings in MDDS [12], were also observed. Except for cholecystitis, these findings indicated circulatory failure, consistent with acute renal failure, as suggested by the serum biochemistry results.

In case 2, numerous diffusely distributed vacuoles of various sizes were observed in the propria mucosae and submucosa of the stomach and duodenum and in the jejunum (Fig. 2c). In the liver, bacteremia with numerous rods, neutrophil infiltration, and liver cell necrosis around the main trunk of the portal vein were found (Fig. 2f). Circulatory failure due to septic shock was suspected from similar findings of liver and kidney congestion in case 1. The presence of neutrophilic infiltration indicated an inflammatory response to septicemia.

## Discussion and conclusions

Here, we present the first report of GE and PI in marmosets. The gross and histopathological findings in both cases matched those of human GE and PI [2, 6, 20]. Numerous vacuoles were histopathologically observed in the stomach and duodenum, and immunohistochemistry confirmed gas cysts. Chronic vomiting had been observed for several years before sudden death. The stomachs and the duodenal segments proximal to the descending region were markedly dilated, accompanied by intra-abdominal fibrous adhesions. Taken together, these findings consistently supported the diagnosis of GE and PI in animals affected by MDDS.

In case 1, gas cysts were distributed in the stomach and duodenum, with gastric ulceration and dilated and congested portal veins. In case 2, the gas cysts reached the jejunum without gastric ulcers, but additional bacteremia was observed. HPVG can occur by high-pressure gas in the GI tract or alteration of the GI mucosa, allowing the gas and intestinal bacteria to enter the portal system through the mesenteric veins [12-14, 18]. Increased intraluminal pressure in MDDSs might lead to emphysema through gas migration into the GI mucosa and submucosa via ulcers and inflow to the portal vein. With these conditions, the animals could have fallen into shock, leading to different outcomes in case 1 (euthanasia) and in case 2 (unexpected death) depending on the site of gas inflow to the portal vein. Therefore, GE and PI in common marmosets constitute fatal conditions in MDDS.

We attempted to identify the etiology of GE in marmosets by considering its etiology in humans**,** with the assumption that GE and PI are the primary lesions in MDDS. Human GE is subclassified into traumatic (mechanical or nonmechanical), obstructive (secondary to malignancy, stricture, volvulus, pyloric stenosis, or duodenal stenosis), and pulmonary (primary) types depending on the etiology [1, 8]. The nonmechanical mucosal traumatic type was the most likely in our cases because its causes included ischemia of the GI tract and gastric dilation in eating disorders [5, 8]. Case 1 showed bloating and vomiting after binge-eating pelleted food and presented with cholecystitis, which is associated with diet [4] and was supposed to be the primary lesion in MDDS [12]. Alterations in the gut microbiome are associated with duodenal stricture [16, 17]. Small intestinal bacterial overgrowth, involving an abnormal increase in the overall bacterial population in the small intestine, could cause gas accumulation in the GI tract. Among the factors influencing the gut microbiome [16], diet was a likely etiology of MDDS in these cases, and GE may have occurred secondary to gastric mucosal damage, similar to what has been observed in human patients [5, 8, 9].

Fatal *Clostridium (C.) perfringens* infections have been reported in marmosets [18, 21]. *C. perfringens* type A toxin was demonstrated in cases of gas gangrene (clostridial myonecrosis) [21] and acute gastric dilation [18], with histopathological findings of not only bacterial enteritis but also subcutaneous, muscle, liver, and lung lesions. Although we did not observe *C. perfringens* infections, these findings did not apply in case 2. Thus, the GE and PI observed in the present cases were unlikely to have occurred as a primary infection [21] or secondary overgrowth [18] of *C. perfringens*.

In conclusion, we report the first cases of simultaneous MDDS, GE, and PI in two captive marmosets. Gross and histopathological findings of gas cysts in the GI tract walls matched the features of human GE and PI. In marmosets with MDDS caused by GE and PI, HPVG and/or secondary bacteremia could lead to hypothermia and shock, causing congestion of the liver and kidney and resulting in death. However, additional cases are needed to determine the prevalence and pathogenesis of GE and PI in MDDSs.

## Data Availability

All the data generated or analyzed during this study are included in this article.

## Abbreviations

BW: Body weight
CLE: Chronic lymphocytic enteritis
GE: Gastric emphysema
GI: Gastrointestinal
HPVG: Hepatic portal venous gas
MDDS: Marmoset duodenal dilation syndrome
PI: Pneumatosis intestinalis
