# Correction: Gastric emphysema and pneumatosis intestinalis in two common marmosets with duodenal dilation syndrome

**DOI:** 10.1186/s12917-024-04117-5

**Published:** 2024-06-12

**Authors:** Shinpei Kawarai, Yasuhiro Sakai, Atsushi Iriki, Yumiko Yamazaki

**Affiliations:** 1 Laboratory for Symbolic Cognitive Development, RIKEN Center for Biosystems Dynamics Research, MI R&D Center Building 3F, 6‑7‑3 Minatojima‑Minamimachi, Chuo‑Ku, Kobe, Hyogo 650‑0047 Japan; 2 Department of Veterinary Nursing for Companion Animals, Chuo Animal General Professional Training College, 1‑12‑17 Tsuji, Shimizu‑ku, Shizuoka, 424‑0806 Japan; 3 Department of Tumor Pathology, Hamamatsu University School of Medicine, 1‑20‑1 Handayama, Chuo‑Ku, Hamamatsu, Shizuoka, 431‑3192 Japan; 4 RIKEN Innovation Design Office, 2‑1 Hirosawa, Wako, Saitama 351‑0198 Japan; 5 Department of Psychological Sciences, Niigata University of Health and Welfare, 1398 Shimami‑Cho, Kita‑Ku, Niigata 950‑3198 Japan

**Correction:** **BMC Vet Res20, 223 (2024)**

10.1186/s12917-024-04087-8

Following publication of the original article [1], the authors would like to update Fig. 2 as they claimed to have used the wrong figure file. As a result, there is a duplication on Figs. 2e and 3a. The correct and incorrect Fig. 2 are given below.

The incorrect Figure:

**Fig. 2:**
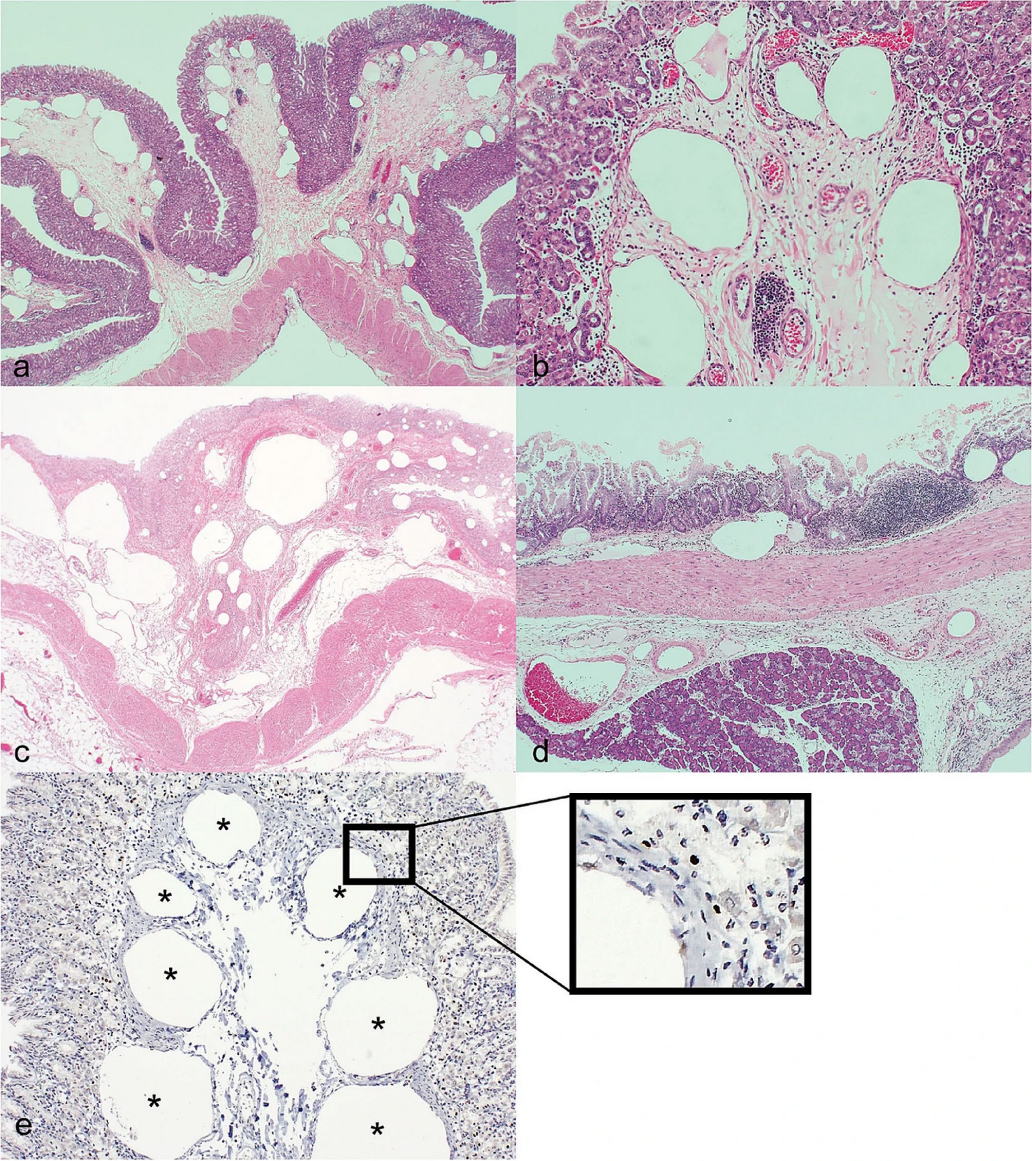
Gastric emphysema and pneumatosis intestinalis, marmoset, HE. **a**, **b** Stomach, case (1) **c** Stomach, case (2) **d** Duodenum, case 1. The gastric and duodenal mucosa contain numerous empty spaces, which are gas cysts. **e** Liver, case (1) Abnormal dilation of the portal vein suggests hepatic portal venous gas. **f** Liver, case (2) There are numerous rods, neutrophil infiltration, and liver cell necrosis around the main trunk of the portal vein

The correct Figure:

**Fig. 2:**
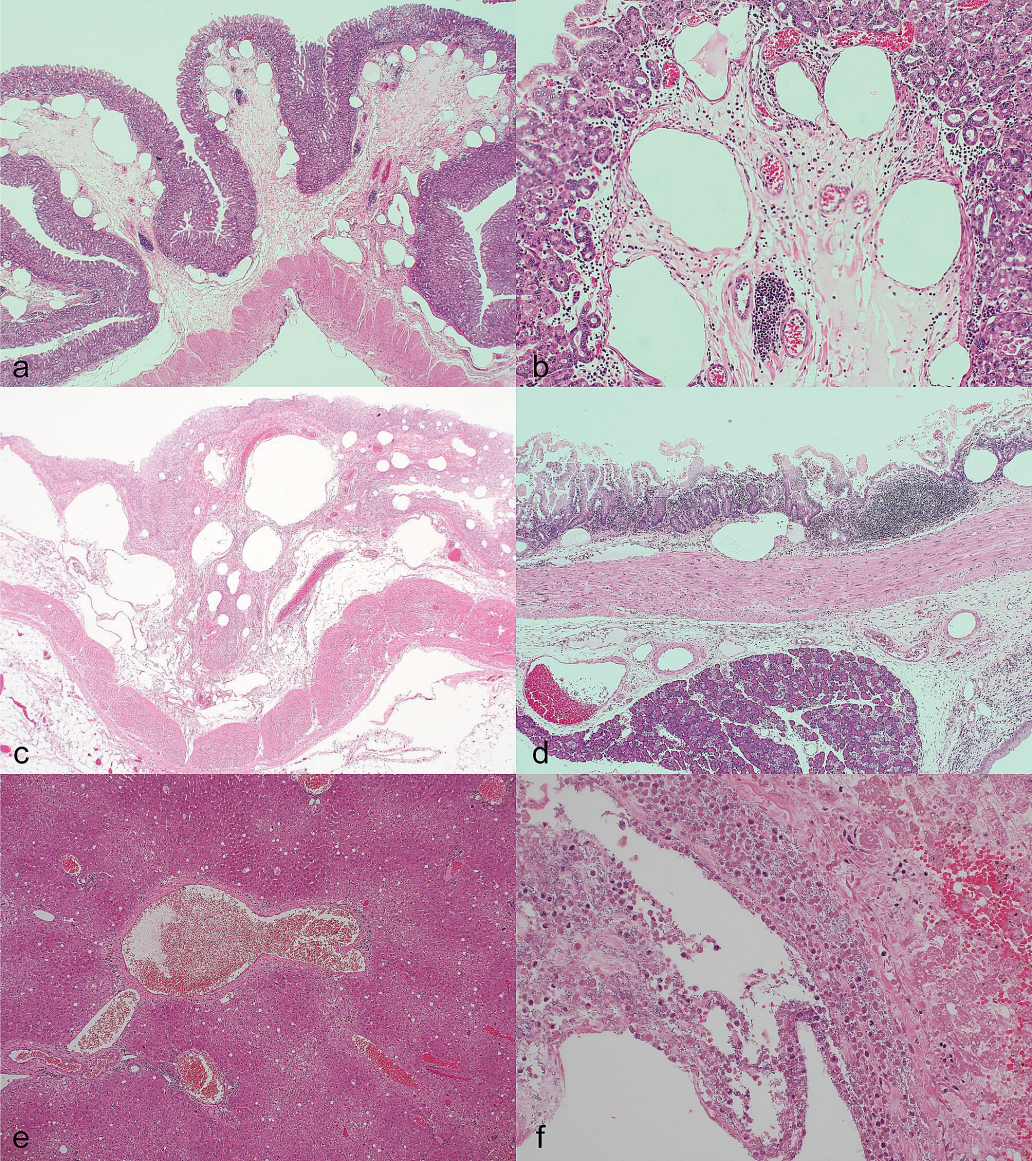
Gastric emphysema and pneumatosis intestinalis, marmoset, HE. **a**, **b** Stomach, case (1) **c** Stomach, case (2) **d** Duodenum, case 1. The gastric and duodenal mucosa contain numerous empty spaces, which are gas cysts. **e** Liver, case (1) Abnormal dilation of the portal vein suggests hepatic portal venous gas. **f** Liver, case (2) There are numerous rods, neutrophil infiltration, and liver cell necrosis around the main trunk of the portal vein
